# Gluten-Free Cereals and Pseudocereals as a Potential Source of Exposure to Toxic Elements among Polish Residents

**DOI:** 10.3390/nu14112342

**Published:** 2022-06-03

**Authors:** Joanna Bielecka, Renata Markiewicz-Żukowska, Anna Puścion-Jakubik, Monika Grabia, Patryk Nowakowski, Jolanta Soroczyńska, Katarzyna Socha

**Affiliations:** Department of Bromatology, Faculty of Pharmacy with the Division of Laboratory Medicine, Medical University of Białystok, Mickiewicza 2D Street, 15-222 Białystok, Poland; joanna.bielecka@umb.edu.pl (J.B.); anna.puscion-jakubik@umb.edu.pl (A.P.-J.); monika.grabia@umb.edu.pl (M.G.); patryk.nowakowski@umb.edu.pl (P.N.); jolasor@interia.pl (J.S.); katarzyna.socha@umb.edu.pl (K.S.)

**Keywords:** buckwheat, millet, maize, quinoa, oat, gluten-free grains, health risk, toxic elements, quality assessment

## Abstract

Background: Nutritional food quality plays a crucial role in maintaining human health. However, food and drinking water, along with occupational exposure, are the main routes of exposure to toxic elements for humans. The main aim of this study was to determine the content of As, Cd, Pb and Hg in naturally gluten-free grains and products made from buckwheat, millet, maize, quinoa and oat. The safety of consumption of the products tested was also assessed. Methods: The contents of As, Cd and Pb were determined using inductively coupled plasma mass spectrometry (ICP-MS). To measure Hg, an atomic absorption spectrometry method (AAS) with the amalgamation technique was applied. To assess the level of consumption of the tested products, an online survey was conducted. To estimate health risk, three indicators were used: the target hazard quotient (THQ), cancer risk (CR) and hazard index (HI). The research material obtained 242 different samples without replications. Results: The highest average content of As, Cd, Pb and Hg were observed for the following groups of products: oat (10.19 µg/kg), buckwheat (48.35 µg/kg), millet (74.52 µg/kg) and buckwheat (1.37 µg/kg), respectively. For six samples, exceedance of established limits was found—three in the case of Cd and three of Pb. Due to the lack of established limits, As and Hg content of the tested products was not compared. Generally, no increased health risks were identified. Conclusions: Based on the obtained results, the consumption of gluten-free cereals and pseudocereals available on the Polish market seems to be safe. However, there is a great need to establish maximum levels of the toxic elements, especially As and Hg in cereal products in European legislation.

## 1. Introduction

Healthy eating and physical activity are key factors in maintaining human health. A proper diet should be based on minimally processed foods, which characterize similar nutritional values as the base material. Unprocessed foods, contrary to processed products, were found to have a low impact on glycemic response and greater satiety potential [1]. Sadler et al. have identified four main aspects of food processing: the extent of change (from the natural state), the nature of changes (e.g., addition food additives), place of processing (who and where it was done) as well as the purpose of processing. It seems to be rational to take all the mentioned aspects into consideration when assessing which products should be considered as processed [2]. Based on the classification made by Sadler et al., cereal and pseudocereal products can be categorized as unprocessed foods. Generally, they are characterized by a high content of carbohydrates, plant proteins, dietary fiber (especially whole grains) and also vitamins and minerals.

Buckwheat is a pseudocereal and belongs to the *Polygonaceae* family. Currently, two main species—common buckwheat (*Fagopyrum esculentum* Moench) and Tartary buckwheat (*Fagopyrum tataricum* Gaertn)—are cultivated and consumed worldwide. Buckwheat is characterized by high protein content with balanced amino acid composition, high content of minerals (e.g., magnesium, zinc and iron) and other biologically active phytochemicals (such as rutin) as well as dietary fiber [3]. It was observed that buckwheat consumption had a positive effect on the lipid profile—lowering the levels of total cholesterol, triglycerides and low-density lipoproteins and increasing the levels of high-density lipoproteins [4]. Consequently, the risk of occurrence of type 2 diabetes mellitus, cancer and cardiovascular disease was reduced [5]. 

Millet is the sixth most important cereal in the world. Pearl millet (*Pennisetum glaucum* (L.)) is the most commonly grown millet species, followed by foxtail millet (*Setaria italica*), proso millet (*Panicum miliaceum*) and finger millet (*Eleusine coracana*) [6]. Millet has a high nutritional value, contains plant protein with a balanced amino acid profile and fiber, and is a good source of micronutrients such as iron and zinc. It has the highest calcium content among all cereals (344 mg/100 g). Moreover, these grains have a low glycemic index; thus, it can be consumed by patients with insulin resistance [6]. Epidemiological data indicate that millet intake could be related to a lower risk of chronic diseases such as obesity and type 2 diabetes mellitus [7]. 

Maize, also called corn, is more often used for feed than for food in Europe; however, there are many products and dishes produced based on this crop. Many types of corn are actually cultivated, and one of the important differences between the selected species is the color of the grain (white, yellow, red and black). Maize is a rich source of carbohydrates, antioxidants (such as carotenoids and phenolic compounds, especially in colored species), minerals (potassium and sodium), B vitamins (B_1_, B_2_, B_3_) and plant protein (with a high content of methionine and cysteine) [8]. 

Oat (*Avena Sativa* (L.)), as well as quinoa, is included in the group of functional grains. In this group, there are also classified lentils, chickpeas, amaranth, chia seeds, wild rice and flaxseeds. Generally, grains in this group are rich in protein, vitamins and minerals compared to white rice, barley and wheat. Oats contain 5–12% fat (75–80% unsaturated), which is higher than other cereals. Moreover, it contains great amounts of dietary fiber, especially β-glucan, which has the ability to lower cholesterol levels and has anti-diabetic properties. This grain is also a rich source of polyphenols such as caffeic, gallic, coumaric and vanillic acids [9]. 

Quinoa (*Chenopodium quinoa* Willd) is characterized by high protein content and quality and is rich in lysine and methionine. The great nutritional value of this pseudocereal is due to considerable amounts of fiber, vitamins (especially B_6_ and B_9_), minerals (such as iron and calcium) and antioxidants. The most common color of grains is white; however, grey, black, yellow and red species are also possible. Quinoa consumption has a positive effect on human health, in particular on gastrointestinal, metabolic and cardiovascular functions [10]. 

Plants are very sensitive to environmental changes, especially stress factors, which include, among others, the influence of toxic elements. Arsenic (As), cadmium (Cd), mercury (Hg) and lead (Pb) are widespread in the environment due to natural processes as well as anthropogenic activities. These elements have no physiological role in the human organism. Even exposure to low levels of these contaminants could be harmful because of their ability to accumulate. Besides occupational exposure, food and drinking water are the main routes of exposure to these toxic elements for humans [11]. Koch et al., in their investigation, showed that cereals accounted for the largest percentage of overall Cd intake through diet among young Polish residents, accounting for 30.2% and 39.6% of the total daily intake of Cd among women and men, respectively. Cereals were also the main contributors of the total Hg intake, which was found to be 15.2% and 16.8% among women and men, respectively [12]. Similar observations were made by Kim et al. in their study. Cereals and bread contributed most (34%) to the overall Cd intake among the American population [13]. A notably higher contribution to the daily Cd intake was observed for cereals and potatoes among Belgian residents (60%) [14]. 

As and Cd were classified by the International Agency for Cancer Research (IARC) in the first group of carcinogens (group 1A) for humans. As exposure may lead to the development of a number of cancers: lung, liver, skin, bladder and kidney, while Cd is related to the occurrence of liver, pancreas, kidney, urinary bladder and breast cancers. Cd exposure is a risk factor for osteoporosis and impairs the functioning of the immune system [15]. 

The IARC categorized Pb in the second group of carcinogens (2A) as probably having a cancerogenic effect on humans. The implications of Pb exposure include impairment in the functioning of kidneys, reproductive and nervous system. Pb is recognized to be a cause of hypertension and a risk factor for heart disease and stroke [15]. 

The target organs in the human body of toxic Hg influence are the brain and central and peripheral nervous system. In addition, this element could impair the functioning of the heart, lungs, immune system and kidneys [16]. 

The main aim of this investigation was to determine the content of toxic elements (As, Cd, Pb and Hg) in naturally gluten-free cereals and pseudocereals available on the Polish market. Moreover, the differences in the contents of the elements between the studied subgroups of the products were checked. Additionally, using a questionnaire survey, the safety of consumption of these products was estimated. 

The novelty of this study includes the analysis of such a broad group of naturally gluten-free products. For the first time, twenty-four subgroups, which consisted of a minimum of five different samples without repetition (each one from a different producer), were taken into account.

## 2. Materials and Methods

### 2.1. Sample Collection

Our study material included samples of five groups: products based on buckwheat (*Fagopyrum* Mill), maize (*Zea mays*), millet (*Panicum miliaceum* L.), oat (*Avena sativa* L.) and quinoa (*Chenopodium quinoa* Willd). Our strategy was to collect as many different types of products as possible to gather the most representative research material for the Polish market. Products were purchased from the local stores in Białystok (a city located in north-eastern Poland) as well as from online stores in April and May 2021. It was also important to obtain products based on the cereals and pseudocereals mentioned above without any additional ingredients. Finally, our research material consisted of 242 samples, and the details of the subgroups and number of samples collected are shown in Table 1, Table 2, Table 3, Table 4 and Table 5. Out of all studied products, 78 were from organic farming. None of the samples were replicated, so each product had a different producer. Moreover, we collected a minimum of 5 samples for every subgroup. Until analyses, products were stored in a cool and dry place in their original packages. 

### 2.2. Microwave Digestion of Samples

To determine the content of As, Cd and Pb, mineral microwave digestion in a close loop system was performed (Berghof, Speedwave, Eningen, Germany). First, samples were homogenized in a stainless-steel mill and weighted (0.2–0.3 g, with an accuracy of 1 mg) to the mineralization polytetrafluoroethylene vessels. Then, 4 mL of concentrated (69%) spectrally pure nitric (V) acid was added (Tracepur, Merck, Darmstadt, Germany). The analytical conditions of microwave digestion were the same as described previously [17]. The digested samples were quantitively transferred into polypropylene vessels with ultra-pure water and stored at −20 °C until further analyses. For the determination of As, Cd and Pb, mineralizates were diluted 10 times with deionized water. 

### 2.3. Toxic Elements Determination

#### 2.3.1. Mercury

Hg content was determined using the atomic absorption spectrometry method (AAS) with the amalgamation technique in a single-purpose atomic absorption spectrometer (Advanced Mercury Analyzer AMA-254, Leco Corp, Altec Ltd., Prague, Czech Republic). Homogenized samples were weighted (100–110 mg, with an accuracy of 0.1 mg) to the cuvette then analyzed using a program dedicated to solid samples. The conditions of the analysis were the same as described previously [17]. 

#### 2.3.2. Arsenic, Cadmium, Lead

To analyze the content of As, Cd and Pb, inductively coupled plasma-mass spectrometry was used (ICP-MS, NexION 300D, PerkinElmer, Waltham, MA, USA). As determination was performed with a kinetic energy discrimination (KED) chamber, while the standard mode was used for Cd and Pb. In the KED mode, polyatomic interferences were corrected through collisions and energy discriminations. Atomic mass units were 74.9216 for As, 109.903, 110.904, 112.905 and 113.904 for Cd, and 205.975, 206.976 and 207.977 for Pb. In both modes (KED and standard), each measurement was replicated 5 times with a dual detector calibration mode. Dwell time per amu and integration time were 50 ms and 1000 ms, respectively. The limits of detection for the methods used were 0.019 μg/kg for As, 0.017 μg/kg for Cd and 0.16 μg/kg for Pb.

#### 2.3.3. Accuracy Check of the Methods

To control the quality of the analyses, certified reference material (CRM) was used (corn flour INCT-CF-3, Institute of Nuclear Chemistry and Technology, Warsaw, Poland). CRM was analyzed at the beginning of the determination as well as every 10 samples. The results of the accuracy check were referred to the values declared by the manufacturer. Recovery for CRM was 101.5%, 99.4%, 100.6% and 99.2% for As, Cd, Pb and Hg, respectively. The precision was 2.4%, 3.4%, 3.1% and 1.9%, respectively.

### 2.4. Participants

An online survey was conducted to assess the level of consumption of the surveyed products among adult Polish residents. Participants were recruited as volunteers through a social media platform between the 5th and 12th of April 2020. The survey was conducted through the Google questionnaire system and was anonymous; no personal data was collected. Each person was allowed to take part in the questionnaire only once. Finally, 168 participants took part in the survey, of which 141 (84%) were women and 27 (16%) were men. The average age of the studied group was 25 years, the average height was 1.69 ± 0.08 m (ranged from 1.5 to 1.99 m) and the average body mass was 65.4 ± 12.3 kg (between 41 and 120 kg). 

#### 2.4.1. Questionnaire

The original questionnaire was included as an attachment to the publication, see supplementary material File S1.

#### 2.4.2. Health Risk Assessment

To assess the safety of the consumption of the studied products, health risk indicators such as target hazard quotient (THQ), hazard index (HI) and cancer risk (CR) were calculated. The following formulas were used:

**THQ** = (FE × DE × LCons × Cx)/(RfD × BW × T) × 10^−3^

**HI** = ∑(THQ As + THQ Cd + THQ Pb + THQ Hg)

**CR** = (FE × DE × EDI × Sf)/T × 10^−3^,

where FE is the frequency of exposure (365 days/year); DE is the duration of exposure (in this work, we have taken the average lifetime of 70 years); LCons is the average level of consumption; Cx is the concentration of the studied element in the sample; RfD is the oral reference dose for As 0.3 µg/kg BW/day, for Cd and Pb 1 µg/kg BW/day, and for Hg 0.3 µg/kg BW/day; BW is the average body weight [18]; T is the overall time of exposure (FE × DE); EDI is the estimated daily intake calculated by multiplying the average daily consumption (g/day) of the selected product and the measured concentration of the element (µg/1000 g); and Sf is the slope factor established by the United States Environmental Protection Agency for As—1.5 mg/kg/day, for Cd—6.3 mg/kg/day, and for Pb—0.0085 mg/kg/day. The reference for the THQ is <1; if the value is higher than 1, it may suggest a potential risk of the toxic elements due to consumption of the studied products, while for the sum of THQ (∑THQ), the limit is 4. The CR estimates the probability of the cancerogenic effect, and values higher than 10^−4^ indicate an increased risk. The average level of daily consumption of the studied products was assessed through an online survey. To estimate the selected health risk indicators, the products with the highest consumption rate such as buckwheat groats, roasted buckwheat groats, millet groats, millet flakes, popcorn and oat flakes were taken into account. The average daily intakes of the above-mentioned products among the study participants were 2.6 g, 3.8 g, 3.9 g, 4.0 g, 3.6 g and 16.6 g, respectively.

### 2.5. Comparison of Results with European Regulations

The obtained results were compared to the Commission Regulation No. 1881/2006, which sets the maximum levels for certain contaminants in foodstuffs. The following maximum levels were set for cereal products: 100 µg/kg for Cd and 200 µg/kg for Pb. Currently, no levels have been set for As and Hg content in cereals other than rice [19]. 

### 2.6. Statistical Analysis

The statistical analyses of the obtained results were performed using Statistica 13 software (TIBCO Software Inc., Palo Alto, CA, USA). First, descriptive statistics were carried out. Parameters such as the average (X), standard deviation (SD), minimum (Min), maximum (Max), median (Me) as well as the lower and upper quartiles (Q_1_–Q_3_) were indicated. To compare the content of the studied elements among the groups and subgroups of products non-parametric tests (Kruskal–Wallis Analysis of Variance (ANOVA) and Mann–Whitney U test) were used. As a significant were considered differences at *p* < 0.05. 

## 3. Results

The results of the analyses conducted in this study are shown in Table 1, Table 2, Table 3, Table 4 and Table 5. In this study, the highest As content was in oat products (10.19 ± 8.12 µg/kg), while the greatest As level was in expanded buckwheat (206.49 µg/kg). However, As concentrations were lower than the limit of detection of the method used. The buckwheat products had the highest average level of Cd (48.35 ± 23.31 µg/kg). Of all the products, considering the individual samples, oat flakes had the lowest Cd level (1.05 µg/kg), in contrast to oat bran, where the maximum Cd concentration was found (138.83 µg/kg). In addition to oat bran, two products—roasted buckwheat (100.60 µg/kg) and buckwheat flour (126.34 µg/kg)—exceeded the maximum levels according to EU legislation. Buckwheat products had the highest average Hg (1.37 ± 1.98 µg/kg). Taking the products into account separately, buckwheat flour (11.50 µg/kg) had higher Hg concentrations than any other product, while the minimum content was in millet flour (0.01 µg/kg). For the last studied element, Pb, the highest content was observed among maize products (74.52 ± 24.02 µg/kg), while one product of millet groats had the highest Pb concentration (436.83 µg/kg). Pb content in this sample was more than two times higher than the established limit, and a similar level was found in buckwheat flour (423.42 µg/kg). The exceedance of the limit was also observed for millet flakes (281.32 µg/kg). On the other hand, in one product of expanded buckwheat, the Pb level was below the limit of detection. 

The health risk was assessed based on the equations described in the material and methods section (point Section 2.4.2). For the THQ, the reference is <1, while for the total THQ (∑THQ), the reference is <4. In our investigation, the assessed values of the indicators were significantly lower than the limits established (Table 6). Similar results were obtained considering CR; the results for each element were lower than 10^−4^.

The results of the statistical analysis are presented in Table 7 and Table 8. The *p*-values were put in the superscript or in the brackets. Many statistically significant differences were found regarding the content of tested elements in the study material, which could indicate that some types of grains have a greater ability to accumulate some heavy metals. No differences were found for Hg content among the tested groups. Among the organic and conventional products, differences were found only for Hg between buckwheat groats (buckwheat groats vs. roasted buckwheat groats).

## 4. Discussion

In this investigation, such a broad group of naturally gluten-free products was analyzed for the first time. The dietary intake of toxic elements such as As, Cd, Pb, and Hg, according to the opinion of the European Food Safety Authority, is a major health care problem [20]. Since 2010, naturally gluten-free grains have gained popularity. The United Nations General Assembly declared 2013 as the ‘International Year of Quinoa’ [21]. In 2020, the value of the global quinoa market amounted to about USD 72 billion. This market is estimated to reach over USD 149 billion by 2026. A higher interest in implementing quinoa into a daily diet is observed especially in Europe and in the US. In 2017, the US buckwheat production volume amounted to about 83 thousand metric tons. Production increased to approximately 86.4 thousand metric tons by 2020 [22]. Naturally gluten-free grains are highly recommended for individuals suffering from gluten-related disorders such as coeliac disease. Laheri and Soon demonstrated that, in a group of patients with coeliac disease, rice, quinoa and maize are the most popular alternative grains. A higher intake of those grains was reported among females [23]. On the other hand, Nikniaz et al. showed inadequate and low whole-grain intake among Azerbaijani-Iranian coeliac patients [24]. It was demonstrated that plants have several protective mechanisms against the accumulation of toxic elements. However, non-essential elements could use different transporters for their transport and uptake into plants. Toxic elements such as Cd and Hg have a negative effect on plant growth and the activity of antioxidant enzymes such as superoxide dismutase [25]. In general, the results of this study indicate that elements such as Cd and Pb tend to accumulate in greater amounts than As and Hg in the studied material. Mitrus and Horbowicz have found that common buckwheat is a type of plant that has a natural ability to accumulate Pb [26]. Taking into account soil contamination, in 2014, approximately 19.4% of the farmland in China was reported as being contaminated by toxic and potentially toxic elements [27]. The regions with the highest content of Cd in the soil were concentrated in Guizhou, Chongqing, Guangxi, Hubei, Hunan and Jiangxi. Generally, areas located in southern China are characterized by higher As, Cd, Pb and Hg content compared to northern regions of China [28]. The anthropometric sources of soil pollution include, among others, usage of chemicals or fertilizers, industrial activities and land application of sewage sludge. On the other hand, natural sources of contaminants include, for example, volcanic eruptions or the release of toxic elements from rocks. Jo and Todorov analyzed the concentration of selected elements in brown rice grains and in bran when 30%, 50%, 70% and 100% of the grain was polished. Even in the first step of polishing (30%), the content of heavy metals was different between the grains and bran (As 221 vs. 598 µg/kg, Cd 14 vs. 23 µg/kg, Hg 4.1 vs. 8.3 µg/kg and Pb 4 vs. 17 µg/kg, respectively). After the complete removal of the bran, the following concentrations were observed: As 205 vs. 599 µg/kg, Cd 12 vs. 22 µg/kg, Hg 3.2 vs. 7.1 µg/kg and Pb 3 vs. 11 µg/kg in the grains and the bran, respectively [29]. To the best of our knowledge, there is no similar research on cereals such as buckwheat, quinoa, maize or oat. In this investigation, six of the samples exceeded the maximum levels established in European legislation—three for Cd and three for Pb. Many statistically significant differences were found in the content of toxic elements between the groups and subgroups tested. 

### 4.1. Arsenic

Food and drinking water, alongside industrial exposure, are the main sources of exposure to As for humans. As levels in food are different depending on the food type, growing conditions (such as type of soil, water, geochemical activity conditions and use of As pesticides) and processing techniques. There is no known physiological function of As for humans. Among cereals, rice has a greater ability to accumulate As and other toxic elements due to its cultivation in flooded soil under reducing conditions. However, there is still limited data regarding the content of As in other types of cereals such as buckwheat, millet, quinoa and oat. In one of the available research papers, the average As content in maize produced in China was 60 µg/kg (30–1300 µg/kg). The results were substantially higher when compared to the results of the present study [30]. However, other Chinese researchers have found that none of the studied maize samples exceeded the established limit for As, and that the average content was 14 µg/kg [31]. In another study, Gu et al. showed that As content in oat and quinoa was 27 µg/kg and 28 µg/kg, respectively [32]. Those results were similar to those obtained in this investigation. A considerably higher As level in quinoa was determined by Bolaños et al. (150 µg/kg) [33].

### 4.2. Cadmium

Cd is widely distributed on the earth and is characterized by a high soil-to-plant transfer and is present in most human foodstuffs. In the study by Lian-Xin et al., Cd levels in buckwheat were higher than in this investigation and ranged from 50 to 285 µg/kg [34]. Lower results were obtained in the research conducted by Gu et al.; the Cd content in oat and quinoa was 13 µg/kg and 49 µg/kg, respectively [32]. A comparable Cd concentration in quinoa grains was reported by Bolaños et al. (40 µg/kg) [33]. Similar results to this work were presented by Bratovcic and Saric; the Cd content in white quinoa grains was 26 µg/kg [35]. Vollmannová et al. reported a higher Cd content in quinoa grains; in their research, the Cd concentration ranged from 90 to 190 µg/kg, while in buckwheat, it was between 50 and 90 µg/kg [36]. Cd levels in maize (100 µg/kg) that were more than ten times higher than obtained in this study (7.11 µg/kg) were determined by Chinese researchers [31].

### 4.3. Lead

The results of research conducted in China showed nearly 1000 times higher Pb content in buckwheat than in this study (from 790 to 4765 µg/kg) [37]. While Turkish researchers reported significantly lower Pb content in buckwheat (190 µg/kg), it was still three times higher than in our research [38]. Additionally, high Pb levels (ranging from 100 to 400 µg/kg) in buckwheat were observed by Slovakian researchers [36]. Considerably high amounts of Pb (1154 µg/kg) were determined in maize available on the Nigerian market [39]. The other research conducted by Larsen et al. also found particularly high Pb levels in Ghanaian maize (2218 µg/kg) and millet (2278 µg/kg) [40]. Other Chinese research showed that the average Pb content in maize was 100 µg/kg, ranging from 20 to 800 µg/kg [30]. In the study conducted by Zheng et al., the average Pb level in maize was 230 µg/kg, and 27% of all the studied samples exceeded the established Pb limits [31]. Similar results to those obtained in this research were shown by Gu et al.; the Pb level in oat was 44 µg/kg, while in quinoa it was 31 µg/kg [32]. In a study conducted by Slovakian researchers, a considerably higher Pb concentration in quinoa was reported—between 330 to 560 µg/kg [36]. In the research carried out by Bolaños et al., the Pb levels in quinoa were lower than the limit of quantification of the method used [33].

### 4.4. Mercury

It has been demonstrated that the Hg content in roots is positively correlated with the Hg content in soil [34]. Similar levels of Hg contamination in maize were found in the investigation carried out by Peng et al. The average Hg content was 1.61 µg/kg, ranging from 1.25 to 2.25 µg/kg [30]. In the investigation carried out by Gu et al., the Hg content in oat and quinoa samples was below the limit of detection of the applied method [32]. The Hg content in maize grown in areas surrounding coal-fired power plants ranged from low levels of 0.55 to nearly forty times higher (21.02 µg/kg) [41]. Zheng et al. determined average Hg content in maize samples comparable with this research (1.4 µg/kg) [31]. A summary of the results obtained by other authors is presented in Table 9.

### 4.5. Health Risk

Currently, there are a few studies available concerning the health risk assessment for toxic elements such as As, Cd, Pb and Hg as a result of the consumption of naturally gluten-free grains. In the research by Zheng et al., the values of THQ of As, Cd, Pb and Hg, taking into account the consumption of maize and the other grains among the Chinese population, were 0.049, 1.03, 0.46 and 0.16, respectively. These findings were considerably higher than those observed in this study [31]. Gu et al. assessed the THQ values for oat consumption among the residents of South Korea, and obtained the following values for As, Cd and Pb: 1.4 × 10^−6^, 2.1 × 10^−7^ and 1.8 × 10^−7^ [32]. These results were lower than in this investigation. No increased risk due to maize and millet consumption was also observed among the Nigerian population. The calculated THQ for both grains was lower than 1 [40].

## 5. Conclusions

Based on the obtained results, naturally gluten-free products available on the Polish market seem to be safe to consume. However, the average daily intake of the tested products among the study participants was rather low. The questionnaire survey was essential in determining whether these products were being consumed. In order to assess the health risk more accurately, it seems necessary to conduct an investigation based on a dietary interview. There is also a great need to establish maximum levels of the toxic elements, especially As and Hg in cereal products in European legislation. Monitoring the content of toxic elements in foodstuff should be a key part of food quality assessment. Moreover, for future research, we see a need to carry out an investigation that looks at the combination of grains eaten in various regions and to determine the health risks.

## Figures and Tables

**Table 1:** The content of the toxic elements in buckwheat products.

Type of Product	As µg/kg	Cd µg/kg	Hg µg/kg	Pb µg/kg
	X ± SD (Min–Max)	X ± SD (Min–Max)	X ± SD (Min–Max)	X ± SD (Min–Max)
	Me (Q_1_–Q_3_)	Me (Q_1_–Q_3_)	Me (Q_1_–Q_3_)	Me (Q_1_–Q_3_)
**Buckwheat groats (*n* = 17)**	3.24 ± 3.57 (0–14.77)	55.85 ± 19.10 (27.94–91.84)	0.70 ± 0.44 (0.23–1.67)	34.91 ± 27.50 (4.57–110.32)
	2.54 (0–4.74)	53.60 (43.73–71.04)	0.53 (0.38–0.91)	38.03 (13.65–49.62)
**Expanded buckwheat (*n* = 7)**	44.84 ± 78.04 (0–206.49)	32.44 ± 10.84 (22.22–49.31)	1.44 ± 1.08 (0.85–3.88)	27.02 ± 50.86 (0–141.60)
	4.72 (4.14–88.87)	27.23 (24.02–43.96)	1.12 (0.97–1.15)	9.577 (0.42–15.72)
**Flakes (*n* = 9)**	2.98 ± 2.09 (0–5.84)	45.09 ± 24.22 (10.70–84.33)	1.91 ± 2.80 (0.42–9.34)	28.62 ± 16.03 (11.47–48.27)
	3.54 (1.99–4.65)	48.27 (26.67–52.92)	1.16 (0.94–1.27)	17.02 (15.82–43.99)
**Flour (*n* = 12)**	3.77 ± 4.69 (0–17.12)	43.21 ± 30.91 (0–126.34)	1.74 ± 3.20 (0.33–11.50)	66.70 ± 114.69 (14.69–423.42)
	3.79 (0–4.75)	32.00 (22.82–52.36)	0.56 (0.45–63.87)	19.28 (17.69–63.97)
**Pasta (*n* = 7)**	7.82 ± 7.75 (2.37–24.42)	37.22 ± 12.17 (22.55–56.55)	1.22 ± 0.64 (0.43–2.40)	25.91 ± 14.72 (14.57–47.85)
	5.11 (2.44–9.92)	35.12 (28.93–50.26)	1.14 (0.82–1.63)	18.58 (17.04–46.84)
**Roasted buckwheat groats (*n* = 9)**	2.77 ± 2.29 (0–5.28)	65.36 ± 20.52 (33.57–100.60)	1.67 ± 2.06 (0.29–6.05)	15.56 ± 15.59 (4.12–56.59)
	4.16 (0–4.71)	64.48 (61.70–74.77)	0.68 (0.33–2.20)	11.89 (10.23–11.99)
**Total (*n* = 61)**	8.53 ± 28.28 (0–206.49)	48.35 ± 23.31 (10.70–126.34)	1.37 ± 1.98 (0.23–11.50)	35.44 ± 56.93 (0–423.42)
	4.13 (1.82–4.90)	45.62 (28.93–61.70)	0.85 (0.46–1.17)	17.04 (11.99–46.84)

X—average; SD—standard deviation; Me—median; Q_1_—lower quartile; Q_3_—upper quartile.

**Table 2:** The content of the toxic elements in maize products.

Type of Product	As µg/kg	Cd µg/kg	Hg µg/kg	Pb µg/kg
	X ± SD (Min–Max)	X ± SD (Min–Max)	X ± SD (Min–Max)	X ± SD (Min–Max)
	Me (Q_1_–Q_3_)	Me (Q_1_–Q_3_)	Me (Q_1_–Q_3_)	Me (Q_1_–Q_3_)
**Cakes (*n* = 9)**	5.43 ± 6.58 (0–21.68)	11.20 ± 11.66 (3.43–39.76)	1.02 ± 0.63 (0.30–2.36)	73.70 ± 6.78 (66.0–87.64)
	2.55 (2.20–7.40)	7.12 (5.26–8.34)	0.90 (0.57–1.16)	74.53 (68.81–75.96)
**Maize groats (*n* = 10)**	10.27 ± 9.03 (2.40–30.68)	35.70 ± 15.32 (21.44–49.19)	0.88 ± 0.76 (0.25–2.45)	56.24 ± 24.62 (39.46–112.57)
	8.37 (4.04–12.22)	33.89 (21.44–49.19)	0.62 (0.31–1.24)	47.70 (40.62–60.65)
**Crisps (*n* = 13)**	2.63 ± 3.04 (0–11.28)	7.32 ± 3.38 (3.57–15.12)	1.80 ± 1.31 (0.43–4.27)	80.80 ± 24.41 (44.56–140.95)
	1.99 (0–3.62)	7.52 (4.62–8.24)	1.22 (0.92–2.42)	75.26 (64.84–89.66)
**Flour (*n* = 11)**	3.39 ± 2.23 (1.66–9.33)	5.30 ± 2.33 (1.66–9.69)	0.62 ± 0.63 (0.20–2.44)	65.04 ± 19.37 (34.92–93.17)
	2.79 (1.78–4.03)	5.53 (3.76–6.60)	0.42 (0.26–0.69)	64.36 (51.35–78.38)
**Pasta (*n* = 8)**	2.48 ± 1.83 (0–5.03)	6.33 ± 4.10 (2.90–15.17)	0.56 ± 0.61 (0.14–2.00)	74.34 ± 13.37 (60.05–96.52)
	2.38 (1.05–3.95)	5.11 (3.40–7.80)	0.40 (0.20–0.57)	70.22 (63.68–85.17)
**Popcorn (*n* = 7)**	4.66 ± 4.38 (0–10.74)	3.68 ± 2.21 (0–6.24)	2.34 ± 0.89 (1.16–3.88)	99.80 ± 22.34 (53.73–117.09)
	3.82 (0–10.36)	4.03 (1.91–5.37)	2.0 (1.92–3.09)	106.65 (88.52–114.70)
**Total (*n* = 59)**	3.63 ± 3.62 (0–21.69)	7.11 ± 7.37 (3.63–7.52)	1.11 ± 1.04 (0.09–4.27)	74.52 ± 24.02 (18.98–140.95)
	2.30 (1.66–4.12)	5.37 (3.62–7.52)	0.71 (0.40–1.47)	73.47 (61.97–87.83)

X—average; SD—standard deviation; Me—median; Q_1_—lower quartile; Q_3_—upper quartile.

**Table 3:** The content of the toxic elements in millet products.

Type of Product	As µg/kg	Cd µg/kg	Hg µg/kg	Pb µg/kg
	X ± SD (Min–Max)	X ± SD (Min–Max)	X ± SD (Min–Max)	X ± SD (Min–Max)
	Me (Q_1_–Q_3_)	Me (Q_1_–Q_3_)	Me (Q_1_–Q_3_)	Me (Q_1_–Q_3_)
**Expanded millet groats (*n* = 6)**	13.82 ± 17.04 (0–40.54)	32.44 ± 10.84 (22.22–49.31)	1.44 ± 1.08 (0.85–3.88)	27.03 ± 50.86 (0–141.60)
	4.72 (4.14–88.87)	27.23 (24.02–43.96)	1.12 (0.97–1.15)	9.56 (0.42–15.72)
**Flakes (*n* = 15)**	4.97 ± 3.29 (2.28–15.95)	35.00 ± 22.23 (7.22–68.80)	0.77 ± 0.64 (0.33–2.61)	58.04 ± 64.43 (20.67–281.32)
	4.39 (3.57–4.57)	25.02 (18.21–59.32)	0.46 (0.40–0.96)	33.07 (26.39–65.91)
**Flour (*n* = 8)**	5.61 ± 4.25 (1.77–14.71)	55.30 ± 25.03 (13.64–76.99)	0.52 ± 0.23 (0.01–0.71)	72.76 ± 22.96 (20.59–91.95)
	4.38 (2.47–7.34)	65.65 (34.65–75.56)	0.58 (0.46–0.68)	76.89 (69.24–88.64)
**Millet groats (*n* = 18)**	4.93 ± 2.24 (0–9.58)	24.55 ± 16.81 (5.70–70.92)	0.64 ± 0.49 (0.12–1.58)	58.48 ± 96.27 (20.67–436.83)
	4.66 (4.19–5.69)	20.74 (10.89–33.89)	0.45 (0.29–1.09)	24.47 (21.66–57.51)
**Total (*n* = 47)**	6.19 ± 6.95 (0–40.54)	33.14 ± 23.92 (5.70–83.71)	0.76 ± 0.61 (0.01–2.77)	57.56 ± 70.10 (19.74–436.83)
	4.55 (3.57–5.69)	22.86 (15.18–53.04)	0.55 (0.40–1.09)	32.90 (23.59–68.15)

X—average; SD—standard deviation; Me—median; Q_1_—lower quartile; Q_3_—upper quartile.

**Table 4:** The content of the toxic elements in oat products.

Type of Product	As µg/kg	Cd µg/kg	Hg µg/kg	Pb µg/kg
	X ± SD (Min–Max)	X ± SD (Min–Max)	X ± SD (Min–Max)	X ± SD (Min–Max)
	Me (Q_1_–Q_3_)	Me (Q_1_–Q_3_)	Me (Q_1_–Q_3_)	Me (Q_1_–Q_3_)
**Bran (*n* = 6)**	10.11 ± 4.93 (5.46–18.27)	60.49 ± 45.64 (6.48–138.83)	1.07 ± 0.39 (0.51–1.66)	56.93 ± 15.96 (38.22–78.62)
	8.29 (6.65–13.69)	50.40 (34.58–68.44)	1.11 (0.83–1.23)	56.23 (43.86–68.43)
**Flakes (*n* = 22)**	10.20 ± 10.00 (1.78–44.11)	27.74 ± 19.32 (1.05–73.22)	1.11 ± 0.85 (0.19–3.57)	47.00 ± 8.88 (18.99–58.26)
	8.12 (2.57–12.84)	21.83 (15.74–31.02)	0.99 (0.42–1.38)	49.14 (18.99–58.26)
**Flour (*n* = 9)**	10.12 ± 3.68 (4.21–15.20)	19.98 ± 9.64 (10.11–41.67)	0.57 ± 0.35 (0.13–1.2)	56.74 ± 13.76 (40.97–86.60)
	10.51 (7.88–12.13)	19.93 (12.67–23.63)	0.47 (0.30–0.80)	55.44 (46.34–61.31)
**Oat groats (*n* = 8)**	10.27 ± 9.04 (2.40–30.68)	24.54 ± 16.81 (5.70–70.92)	0.88 ± 0.76 (0.25–2.45)	56.25 ± 24.63 (39.46–112.58)
	8.37 (0–3.70)	20.74 (21.44–49.19)	0.62 (0.31–1.24)	47.70 (40.62–60.65)
**Total (*n* = 45)**	10.19 ± 8.12 (1.78–44.11)	31.97 ± 24.92 (1.05–138.83)	0.96 ± 0.72 (0.13–3.57)	51.92 ± 0.72 (18.99–112.58)
	8.67 (4.49–12.84)	22.06 (17.94–41.67)	0.83 (0.42–1.20)	49.89 (43.07–55.09)

X—average; SD—standard deviation; Me—median; Q_1_—lower quartile; Q_3_—upper quartile.

**Table 5:** The content of the toxic elements in quinoa grains.

Type of Product	As µg/kg	Cd µg/kg	Hg µg/kg	Pb µg/kg
	X ± SD (Min–Max)	X ± SD (Min–Max)	X ± SD (Min–Max)	X ± SD (Min–Max)
	Me (Q_1_–Q_3_)	Me (Q_1_–Q_3_)	Me (Q_1_–Q_3_)	Me (Q_1_–Q_3_)
**Black quinoa (*n* = 7)**	7.09 ± 2.69 (4.13–9.88)	22.90 ± 5.15 (17.25–32.12)	1.54 ± 0.79 (0.44–2.45)	54.04 ± 8.80 (34.26–58.37)
	8.66 (4.28–9.28)	22.69 (18.33–25.92)	1.86 (0.58–2.13)	57.71 (55.28–58.28)
**Red quinoa (*n* = 7)**	6.86 ± 2.22 (4.09–9.33)	24.57 ± 5.56 (17.22–31.10)	1.58 ± 0.68 (0.81–2.70)	53.60 ± 8.38 (34.75–58.35)
	7.80 (4.64–9.04)	24.95 (17.73–30.66)	1.57 (0.90–2.05)	56.39 (55.30–57.62)
**Tricolor quinoa (*n* = 5)**	9.10 ± 4.02 (5.31–15.95)	27.88 ± 4.55 (5.31–15.95)	0.72 ± 0.67 (0.34–1.92)	55.50 ± 1.12 (54.25–57.30)
	8.13 (7.65–8.45)	8.13 (7.65–8.45)	0.42 (0.41–0.49)	55.26 (55.07–55.59)
**White quinoa (*n* = 11)**	9.94 ± 10.07 (1.98–38.15)	32.20 ± 11.86 (10.03–53.44)	0.55 ± 0.19 (0.24–0.99)	53.46 ± 8.31 (37.69–65.62)
	7.46 (4.10–10.05)	33.72 (24.69–41.01)	0.52 (0.47–0.61)	54.55 (53.76–55.42)
**Total (*n* = 30)**	8.41 ± 6.45 (1.98–38.15)	27.53 ± 8.88 (10.03–53.44)	1.05 ± 0.73 (0.24–2.70)	53.97 ± 7.42 (34.26–65.62)
	7.90 (4.32–9.28)	25.50 (22.69–32.12)	0.70 (0.48–1.86)	55.36 (54.33–57.62)

X—average; SD—standard deviation; Me—median; Q_1_—lower quartile; Q_3_—upper quartile.

**Table 6:** Health risk assessment.

Type of Product	Target Hazard Quotient (THQ)				
	As	Cd	Hg	Pb	∑THQ ^1^
Buckwheat groats	4.03 × 10^−4^	2.09 × 10^−3^	1 × 10^−4^	3.7 × 10^−4^	2.95 × 10^−3^
Roasted buckwheat groats	4.96 × 10^−4^	3.51 × 10^−3^	3 × 10^−4^	2.4 × 10^−4^	4.55 × 10^−3^
Millet groats	9.21 × 10^−4^	1.38 × 10^−3^	1.2 × 10^−4^	9.4 × 10^−4^	3.35 × 10^−3^
Millet flakes	9.37 × 10^−4^	1.98 × 10^−3^	1.5 × 10^−4^	9.4 × 10^−4^	4 × 10^−3^
Popcorn	7.95 × 10^−4^	1.9 × 10^−4^	4 × 10^−4^	1.46 × 10^−3^	2.84 × 10^−3^
Oat flakes	8.08 × 10^−3^	6.59 × 10^−3^	8.8 × 10^−4^	3.10 × 10^−3^	1.87 × 10^−2^
**Cancer Risk (CR)**	3.7 × 10^−7^	6.8 × 10^−5^	-	1.5 × 10^−7^	-

^1^ ∑ = (THQ As) + (THQ Cd) + (THQ Hg) + (THQ Pb).

**Table 7:** Statistically significant differences between the groups of products tested.

Group of Products	Maize	Millet	Quinoa	Oat
**Buckwheat**	Cd ^0.001^, Pb ^0.001^	Cd ^0.01^, Pb ^0.001^	As ^0.001^, Cd ^0.05^, Pb ^0.001^	As ^0.001^, Cd ^0.01^, Pb ^0.001^
**Maize**	-	As ^0.01^, Cd ^0.001^, Pb ^0.001^	As ^0.001^, Cd ^0.001^, Pb ^0.05^	As ^0.001^, Cd ^0.001^, Pb ^0.001^
**Millet**	As ^0.01^, Cd ^0.001^, Pb ^0.001^	-	-	As ^0.05^

**Table 8:** Statistically significant differences between the subgroups of products tested.

As	Cd	Pb	Hg
Buckwheat groats-Oat groats (0.05)	Buckwheat groats-Millet groats (0.01)	Expanded buckwheat-Expanded millet (0.05)	Buckwheat pasta-Corn pasta (0.05)
Corn groats-Oat groats (0.05)	Buckwheat groats-Corn groats (0.001)	Buckwheat flakes-Oat flakes (0.05)	White quinoa-Red quinoa (0.05)
Buckwheat flakes-Oat flakes (0.05)	Corn groats-Oat groats (0.05)	Buckwheat pasta-Corn pasta (0.01)	Conventional buckwheat groats-Organic buckwheat groats (0.05)
Buckwheat pasta-Corn pasta (0.05)	Buckwheat pasta-Corn pasta (0.01)	Buckwheat flour-Millet flour (0.05)	
Buckwheat flour-Oat flour (0.01)	Buckwheat flour-Corn flour (0.001)		
Corn flour-Oat flour (0.01)	Millet flour-Corn flour (0.001)		

*p*-values in brackets.

**Table 9:** Results obtained by other authors.

As	Cd	Hg	Pb
Maize: 60 µg/kg (30–1300 µg/kg) China [30]	Buckwheat: 50–285 µg/kg China [34]	Maize: 1.61 µg/kg (1.25–2.25 µg/kg) China [30]	Maize: 100 µg/kg (20 to 800 µg/kg) China [30]
Maize: 14 µg/kg China [31]	Maize: 100 µg/kg China [31]	Maize: 1.4 µg/kg China [31]	Maize: 230 µg/kg China [31]
Quinoa: 27 µg/kg Oat: 28 µg/kg Korea [32]	Quinoa: 49 µg/kg Oat: 13 µg/kg Korea [32]	Oat: <LOD Quinoa: <LOD Korea [32]	Oat: 44 µg/kg Quinoa: 31 µg/kg Korea [32]
Quinoa: 150 µg/kg Argentina [33]	Quinoa: 40 µg/kg Argentina [33]	Maize: 0.55- 21.02 µg/kg China [41]	Quinoa: <LOQ Argentina [33]
	Quinoa:26 µg/kg [35]		Maize: 2218 µg/kg Millet: 2278 µg/kg Ghana [40]
	Quinoa: 90–190 µg/kg Buckwheat: 50–90 µg/kg Slovakia [36]		Quinoa: 330–560 µg/kg Buckwheat: 100–400 µg/kg Slovakia [36]
			Buckwheat: 190 µg/kg Turkey [38]
			Buckwheat: 790–4765 µg/kg China [34]
			Maize: 1154 µg/kg Nigeria [39]

LOD—limit of detection; LOQ—limit of quantification.

## Data Availability

The data is available from the authors.

## Supplementary Materials

The following supporting information can be downloaded at: https://www.mdpi.com/article/10.3390/nu14112342/s1, File S1: questionnaire.
