# Supplementary material for: Gluten-Free Cereals and Pseudocereals as a Potential Source of Exposure to Toxic Elements among Polish Residents
Source: Nutrients. 2022 Jun 3;14(11):2342. doi: 10.3390/nu14112342 (PMC9182656; doi:10.3390/nu14112342)
Supplement: Supplementary file 1 [file nutrients-14-02342-s001.zip › nutrients-1750787-supplementary.pdf]

File S1

## General questions

1. Age:
2. Sex:
    a. Female
    b. Male
3. Height:
4. Weight:

## Section 1: Buckwheat products

1. Do you consume buckwheat products? If no, please go to the next section.
    a. Yes
    b. No
2. Please check the appropriate answer.

| Type of product | The frequency of consumption | | | | | | |
|---|---|---|---|---|---|---|---|
| | I do not consume | A few times a day | Once a day | A few times a week | Once a week | Once a month | Less than once a month |
| Roasted buckwheat groats | | | | | | | |
| Buckwheat groats | | | | | | | |
| Buckwheat pasta | | | | | | | |
| Buckwheat flour | | | | | | | |
| Buckwheat flakes | | | | | | | |
| Expanded buckwheat groats | | | | | | | |

3. Please estimate the quantity of buckwheat products consumed at one time (the quantity refers to the raw product; 1 portion of the product= 50 g or ½ bag).

| Type of product | The portion size | | | | | |
|---|---|---|---|---|---|---|
| | Not applicable | 1/2 portion | 1 portion | 2 portions | 3 portions | 4 portions and more |
| Roasted | | | | | | |

| | | | | | | |
|---|---|---|---|---|---|---|
| buckwheat groats | | | | | | |
| Buckwheat groats | | | | | | |
| Buckwheat flakes | | | | | | |
| Buckwheat flour | | | | | | |

4. Please estimate the quantity of buckwheat pasta consumed at one time (the quantity refers to the raw product; 1 portion of the product= 1 glass= 80 g).

| Type of product | The portion size | | | | | |
|---|---|---|---|---|---|---|
| | Not applicable | 1/2 portion | 1 portion | 2 portions | 3 portions | 4 portions and more |
| Buckwheat pasta | | | | | | |

5. Please estimate the quantity of expanded buckwheat groats consumed at one time (the quantity refers to the raw product; 1 portion of the product= one glass= 15 g).

| Type of product | The portion size | | | | | |
|---|---|---|---|---|---|---|
| | Not applicable | 1/2 portion | 1 portion | 2 portions | 3 portions | 4 portions and more |
| Expanded buckwheat groats | | | | | | |

## **Section 2: Millet products**

1. Do you consume millet products? If no, please go to the next section.
   a. Yes
   b. No
2. Please check the appropriate answer.

| Type of product | The frequency of consumption | | | | | | |
|---|---|---|---|---|---|---|---|
| | I do not consume | A few times a day | Once a day | A few times a week | Once a week | Once a month | Less than once a month |
| Millet groats | | | | | | | |

| | | | | | | | |
|---|---|---|---|---|---|---|---|
| Millet flour | | | | | | | |
| Millet flakes | | | | | | | |
| Expanded millet groats | | | | | | | |

3. Please estimate the quantity of millet products consumed at one time (the quantity refers to the raw product; 1 portion of the product= 50 g or ½ bag).

| Type of product | The portion size | | | | | |
|---|---|---|---|---|---|---|
| | Not applicable | 1/2 portion | 1 portion | 2 portions | 3 portions | 4 portions and more |
| Millet groats | | | | | | |
| Millet flour | | | | | | |
| Millet flakes | | | | | | |

4. Please estimate the quantity of expanded millet consumed at one time (the quantity refers to the raw product; 1 portion of the product= 1 glass= 15 g).

| Type of product | The portion size | | | | | |
|---|---|---|---|---|---|---|
| | Not applicable | 1/2 portion | 1 portion | 2 portions | 3 portions | 4 portions and more |
| Expanded millet groats | | | | | | |

## Section 3: Quinoa

1. Do you consume quinoa grains? If no, please go to the next section.
    a. Yes
    b. No
2. Please check the appropriate answer.

| Type of product | The frequency of consumption | | | | | | |
|---|---|---|---|---|---|---|---|
| | I do not consume | A few times a day | Once a day | A few times a week | Once a week | Once a month | Less than once a month |
| White quinoa | | | | | | | |
| Black quinoa | | | | | | | |

| | | | | | | | |
|---|---|---|---|---|---|---|---|
| Red quinoa | | | | | | | |
| Tricolor quinoa | | | | | | | |

3. Please estimate the quantity of quinoa consumed at one time (the quantity refers to the raw product; 1 portion of the product= 1/2 bag= 50 g).

| Type of product | The portion size | | | | | |
|---|---|---|---|---|---|---|
| | Not applicable | 1/2 portion | 1 portion | 2 portions | 3 portions | 4 portions and more |
| White quinoa | | | | | | |
| Black quinoa | | | | | | |
| Red quinoa | | | | | | |
| Tricolor quinoa | | | | | | |

**Section 4: Corn products**

1. Do you consume corn products? If no, please go to the next section.
    a. Yes
    b. No
2. Please check the appropriate answer.

| Type of product | The frequency of consumption | | | | | | |
|---|---|---|---|---|---|---|---|
| | I do not consume | A few times a day | Once a day | A few times a week | Once a week | Once a month | Less than once a month |
| Corn groats | | | | | | | |
| Corn flour | | | | | | | |
| Corn pasta | | | | | | | |
| Corn crisps | | | | | | | |
| Corn cakes | | | | | | | |
| Popcorn | | | | | | | |

3. Please estimate the quantity of corn products consumed at one time (the quantity refers to the raw product; 1 portion of the product= 50 g or ½ bag).

| Type of product | The portion size | | | | | |
|---|---|---|---|---|---|---|
| | Not applicable | 1/2 portion | 1 portion | 2 portions | 3 portions | 4 portions and more |
| Corn groats | | | | | | |
| Corn flour | | | | | | |

4. Please estimate the quantity of corn pasta consumed at one time (the quantity refers to the raw product; 1 portion of the product= 1 glass= 80 g).

| Type of product | The portion size | | | | | |
|---|---|---|---|---|---|---|
| | Not applicable | 1/2 portion | 1 portion | 2 portions | 3 portions | 4 portions and more |
| Corn pasta | | | | | | |

5. Please estimate the quantity of expanded corn products consumed at one time (1 portion of the crisps or popcorn= 1 handful; 1 portion of cakes= 1 cake).

| Type of product | The portion size | | | | | |
|---|---|---|---|---|---|---|
| | Not applicable | 1/2 portion | 1 portion | 2 portions | 3 portions | 4 portions and more |
| Corn crisps | | | | | | |
| Corn cakes | | | | | | |
| Popcorn | | | | | | |

**Section 5: Oat products**

1. Do you consume oat products? If no, please go to the next section.
   a. Yes
   b. No
2. Please check the appropriate answer.

| Type of product | The frequency of consumption | | | | | | |
|---|---|---|---|---|---|---|---|
| | I do not consume | A few times a day | Once a day | A few times a week | Once a week | Once a month | Less than once a month |
| Oat flakes | | | | | | | |
| Oat flour | | | | | | | |
| Oat bran | | | | | | | |
| Oat groats | | | | | | | |

3. Please estimate the quantity of oat products consumed at one time (the quantity refers to the raw product; 1 portion of the product= 50 g or ½ bag).

| Type of product | The portion size | | | | | |
|---|---|---|---|---|---|---|
| | Not applicable | 1/2 portion | 1 portion | 2 portions | 3 portions | 4 portions and more |
| Oat flakes | | | | | | |
| Oat groats | | | | | | |
| Oat flour | | | | | | |

4. Please estimate the quantity of oat bran consumed at one time (1 portion of the product= 1 spoon = 7 g).

| Type of product | The portion size | | | | | |
|---|---|---|---|---|---|---|
| | Not applicable | 1/2 portion | 1 portion | 2 portions | 3 portions | 4 portions and more |
| Oat bran | | | | | | |
